# CD109 Promotes Drug Resistance in A2780 Ovarian Cancer Cells by Regulating the STAT3-NOTCH1 Signaling Axis

**DOI:** 10.3390/ijms241210306

**Published:** 2023-06-18

**Authors:** Jun Se Kim, Min Joo Shin, Seo Yul Lee, Dae Kyoung Kim, Kyung-Un Choi, Dong-Soo Suh, Dayea Kim, Jae Ho Kim

**Affiliations:** 1 Department of Physiology, School of Medicine, Pusan National University, Yangsan 50612, Republic of Korea; wnstp93@naver.com (J.S.K.);; 2 Hicelltech Inc., Yangsan 50612, Republic of Korea; 3 Department of Pathology, School of Medicine, Pusan National University, Yangsan 50612, Republic of Korea; 4 Department of Obstetrics and Gynecology, School of Medicine, Pusan National University, Yangsan 50612, Republic of Korea; 5 New Drug Development Center, Daegu-Gyeongbuk Medical Innovation Foundation (K-MEDI hub), Daegu 41061, Republic of Korea; dayea@dgmif.re.kr

**Keywords:** epithelial ovarian cancer, A2780, drug resistance, cluster of differentiation 109 (CD109), signal transducer and activator of transcription 3 (STAT3), neurogenic locus notch homolog protein 1 (NOTCH1)

## Abstract

Epithelial ovarian cancer (EOC) is the most lethal gynecological malignancy owing to relapse caused by resistance to chemotherapy. We previously reported that cluster of differentiation 109 (CD109) expression is positively correlated with poor prognosis and chemoresistance in patients with EOC. To further explore the role of CD109 in EOC, we explored the signaling mechanism of CD109-induced drug resistance. We found that CD109 expression was upregulated in doxorubicin-resistant EOC cells (A2780-R) compared with that in their parental cells. In EOC cells (A2780 and A2780-R), the expression level of CD109 was positively correlated with the expression level of ATP-binding cassette (ABC) transporters, such as ABCB1 and ABCG2, and paclitaxel (PTX) resistance. Using a xenograft mouse model, it was confirmed that PTX administration in xenografts of CD109-silenced A2780-R cells significantly attenuated in vivo tumor growth. The treatment of CD109-overexpressed A2780 cells with cryptotanshinone (CPT), a signal transducer and activator of transcription 3 (STAT3) inhibitor, inhibited the CD109 overexpression-induced activation of STAT3 and neurogenic locus notch homolog protein 1 (NOTCH1), suggesting a STAT3-NOTCH1 signaling axis. The combined treatment of CD109-overexpressed A2780 cells with CPT and N-[N-(3,5-difluorophenacetyl)-l-alanyl]-S-phenylglycine t-butyl ester (DAPT), a NOTCH inhibitor, markedly abrogated PTX resistance. These results suggest that CD109 plays a key role in the acquisition of drug resistance by activating the STAT3-NOTCH1 signaling axis in patients with EOC.

## 1. Introduction

Epithelial ovarian cancer (EOC) is the leading cause of cancer-related deaths in women, accounting for 90% of ovarian cancer cases [1]. The surgical removal of the tumor mass and platinum- and taxane-based chemotherapy are the current standard treatments and are effective in approximately 70% of patients [2]. However, even with treatment, most patients with advanced ovarian cancer develop drug resistance within 18 months with a 5-year survival rate of only 31.6% due to the relapse and metastasis of drug-resistant cells to sites distant from the ovary, such as lungs or the liver [3,4,5]. Cancer cells acquire drug resistance through various mechanisms, such as drug inactivation, multidrug resistance, the blockage of the apoptosis pathway, alteration in drug metabolism, DNA repair, and enhanced gene amplification [6]. Among these mechanisms, the release of various chemotherapeutic drugs using adenosine triphosphate-binding cassette (ABC) transporters, such as ABCB1 (multidrug resistance protein 1 or P-glycoprotein) and ABCG2 (breast cancer resistance protein), which are adenosine triphosphate (ATP)-dependent drug efflux membrane proteins, is the most common mechanism for developing drug resistance in the ovarian cancer cells [7,8]. Therefore, the molecular mechanisms through which ovarian cancer cells acquire drug resistance must be elucidated to effectively prevent and treat cancer recurrence and metastasis.

The cluster of differentiation 109 (CD109) is a glycosylphosphatidylinositol-anchored cell surface glycoprotein and a member of the α2-macroglobulin-C3, C4, and C5 family of thioester-containing proteins [9]. It was originally identified as a cell surface antigen detected using a monoclonal antibody raised against a myeloid cell line (KG1a) [10]. CD109 is expressed in phytohemagglutinin-activated T cells, thrombin-activated platelets, endothelial cells, a subset of CD34^+^ bone marrow cells that contain megakaryocyte progenitors, and mesenchymal stem cell subsets [11,12,13]. Furthermore, it has been reported that CD109 is highly expressed in various malignant tumors, including lung, liver, pancreas, esophagus, uterus, oral cavity, breast cancer, glioblastoma, and several types of sarcomas, and is associated with a poor prognosis [9]. The elevation of CD109 promotes metastasis and drug resistance in lung cancer by activating the epidermal growth factor receptor (EGFR)–protein kinase B (AKT)–mammalian target of rapamycin (mTOR) signaling pathway [14]. Furthermore, it has been reported that the activation of the signal transducer and activator of transcription 3 (STAT3) through the interaction of CD109 and glycoprotein 130 (GP130) promotes plasticity and chemoresistance in glioblastoma stem cells [15]. A high expression of CD109 correlates with poor prognosis and chemoresistance in patients harboring EOC [16], but the role of CD109 in the drug resistance of ovarian cancer cells and its underlying mechanism remain largely unknown.

In this study, we explored the role of CD109 in the drug resistance of ovarian cancer cells in vitro and in vivo. Moreover, we investigated, for the first time, the role of the STAT3- neurogenic locus notch homolog protein 1 (NOTCH1) signaling axis in CD109-stimulated drug resistance.

## 2. Results

### 2.1. CD109 Expression Is Upregulated in Drug-Resistant A2780 Cells

**Figure 1:**
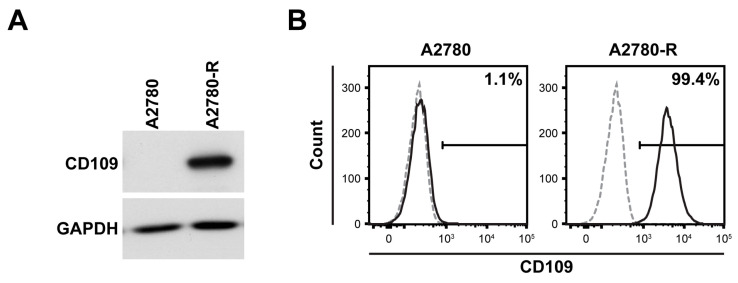
Increased expression of CD109 in drug-resistant A2780 (A2780-R) cells: (**A**) Western blot analysis of CD109 in A2780 and A2780-R cells; (**B**) flow cytometry analysis of CD109 expression in A2780 and A2780-R cells. Flow cytometry histogram overlays show staining for CD109 antibody (black solid lines) and isotype-matched control antibody (gray dotted lines). The percentages of the CD109-expressing population are indicated.

We have previously reported that increased CD109 expression is associated with chemoresistance in patients with EOC using immunohistochemical staining and mRNA expression analysis [16]. Based on previous studies, we compared CD109 expression in the ovarian cancer cell line A2780 and a previously established drug-resistant ovarian cancer cell line (A2780-R) [17]. A2780-R cells exhibited increased ABC transporter protein levels and resistance to anticancer drugs (cisplatin, doxorubicin, and paclitaxel (PT)) compared with A2780 parental cells (Figure S1). Furthermore, the protein level of CD109 was greatly increased in A2780-R cells compared with that in parental cells (Figure 1A). Flow cytometry results indicated that the percentage of CD109-expressing A2780-R cells was estimated to be 99.4% (Figure 1B), suggesting that CD109 is highly expressed in drug-resistant EOC cells.

### 2.2. CD109 Overexpression Increases Drug Resistance of A2780 Cells

**Figure 2:**
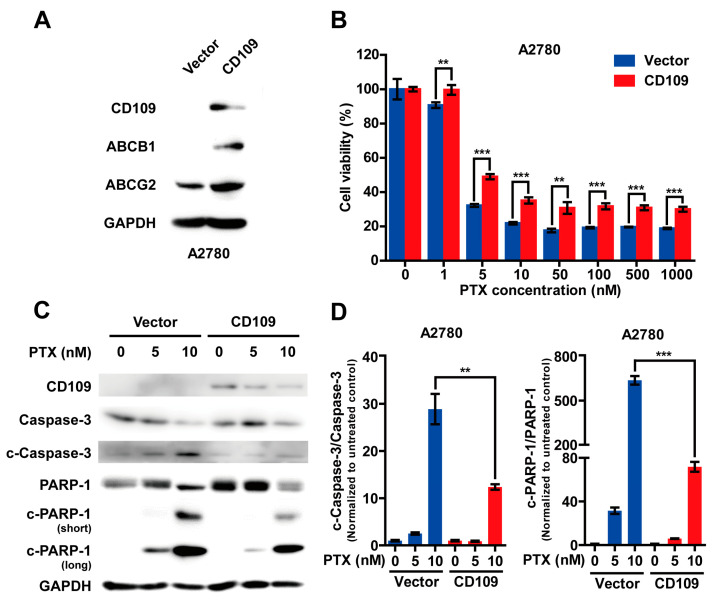
Overexpression of CD109 increases drug resistance in A2780 cells: (**A**) Western blot analysis of CD109 and adenosine triphosphate-binding cassette (ABC) transporters in A2780 control and CD109-overexpressing cells; (**B**) effects of CD109 overexpression on paclitaxel (PTX)-induced cell death in A2780 cells. Data are presented as mean ± SD (** *p* < 0.01, *** *p* < 0.001); (**C**) Western blot analysis of apoptosis markers in A2780 control and CD109-overexpressing cells. After treatment with 0, 5, or 10 nM of PTX for 48 h, cleavage of caspase-3 and PARP-1 was confirmed; (**D**) bar graphs quantifying cleaved caspase-3 (left) and PARP-1 (right) to total caspase-3 and PARP-1, respectively, as assessed using pooled densitometric data. Each bar was normalized to the untreated control (** *p* < 0.01; *** *p* < 0.001).

To investigate whether CD109 regulates drug resistance in A2780 cells, CD109 was overexpressed in A2780 cells via lentiviral infection. CD109-overexpressed A2780 cells showed an increase in ABCB1 and ABCG2 protein expression levels (Figure 2A), which are associated with drug resistance and the poor prognosis of ovarian cancer [18]. In A2780 cells overexpressing CD109, treatment with the ABCB1 inhibitor Elacridar or the ABCG2 inhibitor Ko143 significantly attenuated the resistance of the cells to PTX (Figure S2), suggesting a pivotal role of ABCB1 and ABCG2 in CD109-stimulated drug resistance. PTX treatment dose-dependently reduced the viability of A2780 cells; however, the overexpression of CD109 increased the resistance of A2780 cells to PTX (Figure 2B). The cleavage of poly(ADP-ribose) polymerase (PARP)-1 by cleaved caspase-3 (activated form) is a key step in the apoptosis signaling pathway [19,20]. PTX treatment dose-dependently increased the cleavage of caspase-3, and PARP-1 increased in A2780 cells in a dose-dependent manner; however, CD109 overexpression markedly attenuated the PTX-induced cleavage of caspase-3 and PARP-1 (Figure 2C,D).

### 2.3. CD109 Knockdown Abrogates Drug Resistance of A2780-R Cells

**Figure 3:**
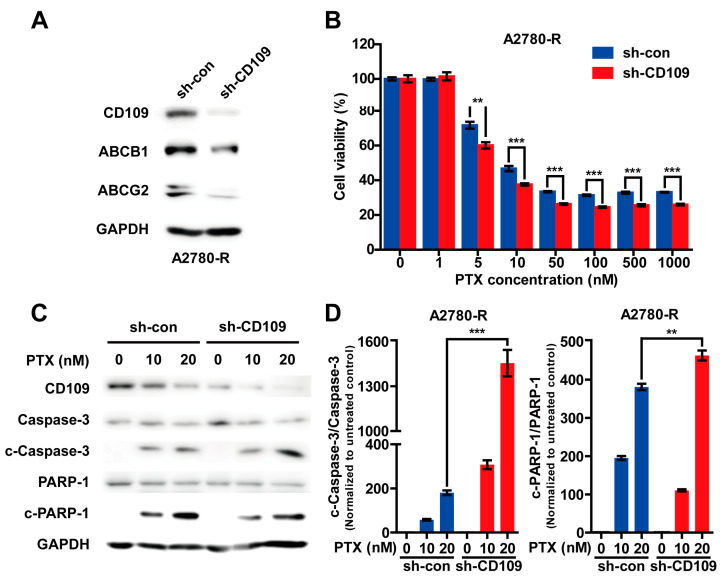
Knockdown of CD109 attenuates drug resistance in A2780-R cells: (**A**) Western blot analysis of CD109 and ABC transporters in A2780-R cells infected with lentiviruses bearing control shRNA (sh-con) and CD109 shRNA (sh-CD109); (**B**) effects of CD109 knockdown on PTX-induced cell death in A2780-R cells. The sh-control and sh-CD109 A2780-R cells were treated with increasing doses of PTX, followed by measurement of cell viability. Data are presented as mean ± SD (** *p* < 0.01; *** *p* < 0.001); (**C**) Western blot analysis of apoptosis markers in A2780-R sh-control and sh-CD109 cells. After treatment with 0, 10, or 20 nM of PTX for 48 h, cleavage of caspase-3 and PARP-1 was confirmed; (**D**) bar graphs quantifying cleaved caspase-3 (**left**) and PARP-1 (**right**) to total caspase-3 and PARP-1, respectively, as assessed using pooled densitometric data. Each bar was normalized to the untreated control (** *p* < 0.01; *** *p* < 0.001).

Given that CD109 overexpression could induce drug resistance in A2780 cells, we next investigated the effect of CD109 knockdown on drug resistance by infecting A2780-R cells with a lentivirus encoding CD109-specific shRNA. CD109 knockdown reduced the protein expression levels of ABC transporters in the A2780-R cells, leading to decreased drug resistance to PTX treatment (Figure 3A,B). Consistently, PTX treatment dose-dependently increased the cleavage of caspase-3 and PARP-1 in the A2780-R cells. However, the silencing of CD109 augmented the levels of cleaved caspase-3 and PARP-1 (Figure 3C,D), supporting the role of CD109 in the drug resistance of A2780 cells.

### 2.4. CD109 Knockdown Abrogates Drug Resistance in the Ovarian Cancer Xenograft Model

**Figure 4:**
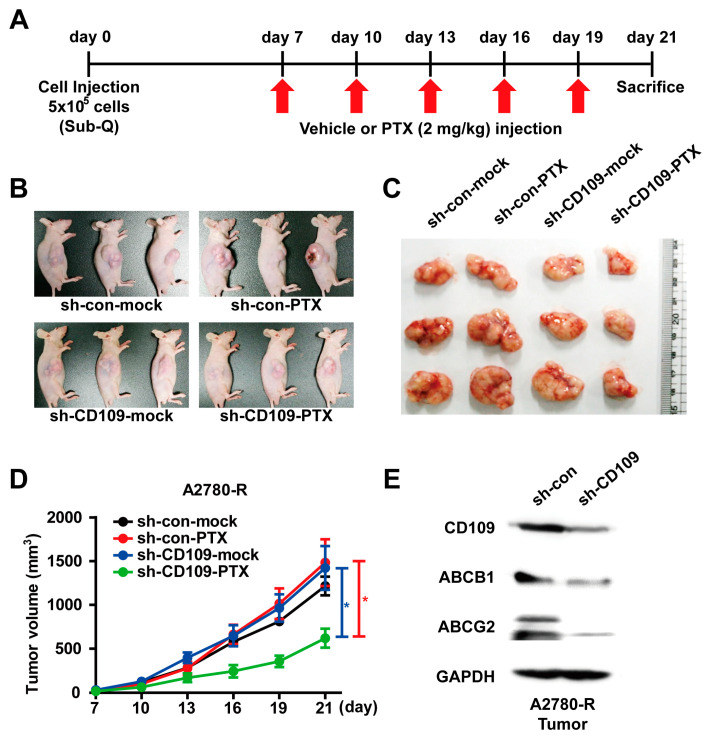
CD109 knockdown attenuates drug resistance of transplanted A2780-R cells in vivo: (**A**) experimental scheme of xenograft tumor model. Vehicle or PTX (2 mg/kg) was injected intraperitoneally every 3 days from 7 days after cell injection. PBS was used as the vehicle. Representative images of nude mice (**B**) and tumors (**C**) on day 21 after injection of cells; (**D**) tumor volume measured at indicated time points after injection of cells (the blue * *p* < 0.05, sh-CD109-mock vs. sh-CD109-PTX; the red * *p* < 0.05, sh-con-PTX vs. sh-CD109-PTX). (**E**) Western blot analysis of CD109 and ABC transporters in A2780-R sh-control and sh-CD109 tumors.

To explore whether the knockdown of CD109 in A2780-R cells attenuated drug resistance in vivo, the A2780-R cells were infected with lentiviruses bearing either CD109 shRNA or control shRNA, followed by subcutaneous injection into the right flank of nude mice. One week after cell transplantation, phosphate-buffered saline (PBS) and PTX (2 mg/kg) were intraperitoneally injected every three days, and the in vivo growth of tumors was measured at different time points (Figure 4A). CD109 silencing did not significantly impact the in vivo growth of A2780-R cells. In contrast, tumor growth was markedly reduced due to PTX treatment in mice injected with sh-CD109 cells (Figure 4B–D). Consistent with the in vitro results, the expression levels of ABC transporters were positively correlated with the expression levels of CD109 in transplanted tumors (Figure 4E). These results indicate that the knockdown of CD109 significantly inhibits tumor growth in vivo by reducing the drug resistance of A2780-R tumors.

### 2.5. CD109 Regulates STAT3 and NOTCH1 Signaling Pathways

**Figure 5:**
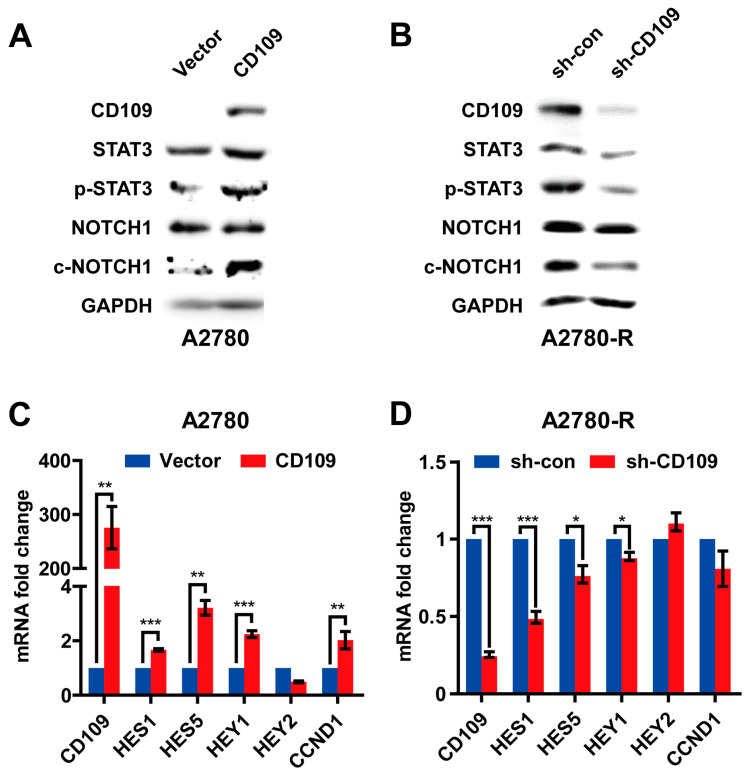
CD109 regulates STAT3 and NOTCH1 signaling in A2780 cells: (**A**) Western blot analysis of STAT3 and NOTCH1 signaling in A2780 cells after CD109 overexpression; (**B**) effects of CD109 knockdown on the STAT3-NOTCH1 signaling axis were measured through Western blot analysis of STAT3 and NOTCH1 signaling. Quantitative real-time PCR analysis of *CD109*, *HES1*, *HES5*, *HEY1*, *HEY2*, and *CCND1* in CD109-overexpressed A2780 cells (**C**) and CD109-silenced A2780-R cells (**D**). Bar graphs represent *CD109*, *HES1*, *HES5*, *HEY1*, *HEY2* and *CCND1* expression levels normalized to *GAPDH* (* *p* < 0.05, ** *p* < 0.01, and *** *p* < 0.001).

Next, we investigated the intracellular signaling pathways that regulate CD109-induced drug resistance in A2780 cells. It has been reported that the CD109/STAT3 signaling axis is a mediator of chemoresistance in glioblastoma stem cells [15]. In addition, not only STAT3 but also the NOTCH1 signaling pathway is known to be involved in the acquisition of chemotherapy resistance in head and neck squamous cell carcinoma (HNSCC), breast cancer, and gastrointestinal cancer [21,22,23]. Therefore, we investigated whether STAT3 and NOTCH1 signals are regulated by CD109 in A2780 cells. The overexpression of CD109 in A2780 parental cells induced STAT3 phosphorylation and NOTCH1 cleavage (Figure 5A). In contrast, the shRNA knockdown of CD109 in A2780-R cells attenuated STAT3 phosphorylation and NOTCH1 cleavage (Figure 5B). To confirm these results, we performed the transient knockdown of CD109 using siRNA. Similar to shRNA-mediated knockdown, the siRNA-mediated silencing of CD109 expression in A2780-R cells also reduced the protein expression of ABC transporters and the activation of STAT3 and NOTCH1 (Figure S3). The mRNA levels of hairy and enhancer of split 1 and 5 (HES1 and 5) and hairy/enhancer-of-split related with YRPW motif protein 1 (HEY1), the target genes of NOTCH1, were increased by CD109 overexpression in A2780 cells and attenuated by CD109 knockdown in A2780-R cells, respectively (Figure 5C,D). These results indicated that CD109 induced the activation of STAT3- and NOTCH1-dependent signals in A2780 cells.

### 2.6. Drug Resistance Is Regulated via CD109-STAT3-NOTCH1 Signaling Axis in A2780 Cells

**Figure 6:**
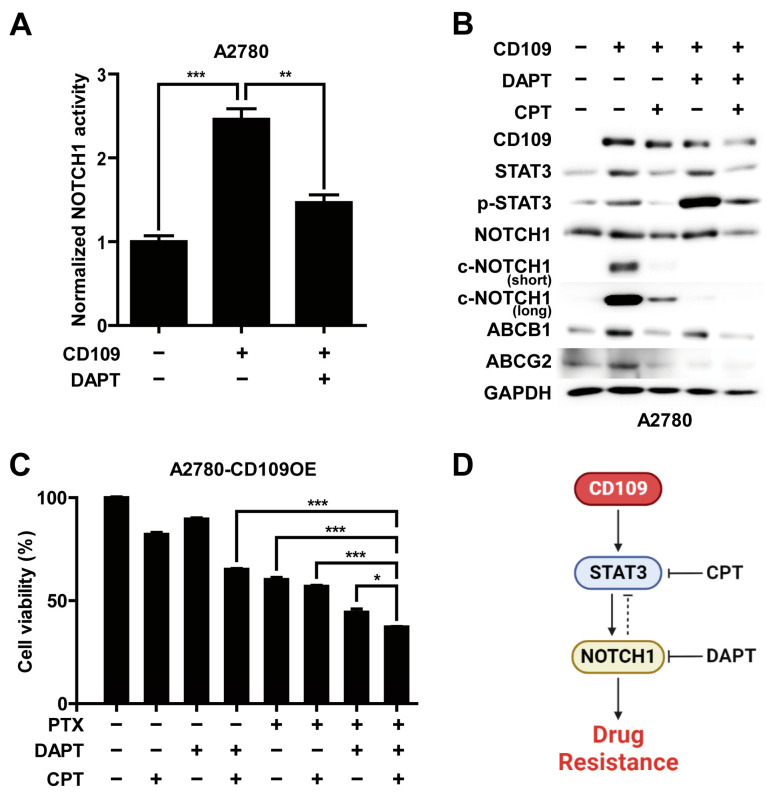
Drug resistance in A2780 cells is regulated via the CD109-STAT3-NOTCH1 signaling axis: (**A**) measurement of NOTCH1 activity by monitoring the amount of luminescence via 4xCSL promoter transcription. A2780 CD109-overexpressing cells were treated with 25 μM of DAPT for 24 h (** *p* < 0.01; *** *p* < 0.001); (**B**) Western blot analysis of STAT3 and NOTCH1 signaling and ABC transporters after single or combined treatment with CPT (2 μM) and DAPT (25 μM) for 24 h in A2780 (control and CD109 overexpressing) cells; (**C**) cell viability assay in which A2780 CD109-overexpressing cells underwent single, dual-, or triple-combinatorial treatment with PTX (5 nM), CPT (2 μM), and DAPT (25 μM) for 24 h (* *p* < 0.05; *** *p* < 0.001); (**D**) schematic representation of the mechanism proposed for CD109 to act as a key regulator of the STAT3-NOTCH1 signaling axis and switch of drug resistance in A2780 cells.

To determine whether STAT3 and NOTCH1 signaling pathways are implicated in CD109-induced drug resistance in ovarian cancer cells, we examined the effects of STAT3 and NOTCH1 inhibitors on CD109-induced drug resistance. Using a Notch-responsive luciferase reporter assay, we measured the Notch-dependent transcriptional activity. In A2780 cells, NOTCH1 activity induced by CD109 overexpression was markedly attenuated by treatment with N-[N-(3,5-difluorophenacetyl)-l-alanyl]-S-phenylglycine t-butyl ester (DAPT), a NOTCH pathway inhibitor (Figure 6A). Next, we investigated the effects of cryptotanshinone (CPT), a STAT3-specific inhibitor, and DAPT on CD109-induced STAT3 and NOTCH1 activity and the protein expression of ABC transporters in the A2780 cells (Figure 6B). The phosphorylation of STAT3, the cleavage of NOTCH1, and the expression of ABC transporters increased in response to CD109 overexpression. CPT treatment significantly inhibited CD109-induced NOTCH1 cleavage and STAT3 phosphorylation in the A2780 cells. Furthermore, DAPT treatment almost completely inhibited the CD109-induced cleavage of NOTCH1; however, it markedly increased STAT3 phosphorylation. In addition, the combined treatment with CPT and DAPT inhibited STAT3 and NOTCH1 activation. CPT and DAPT treatment inhibited the expression of ABC transporters. The expression of ABCB1 was slightly attenuated via DAPT treatment in contrast to the marked downregulation of ABCG2 expression. These results suggest that NOTCH signaling plays a preferential role in the regulation of ABCG2. Notably, the combined treatment with DAPT and CPT more potently suppressed the protein expression of ABC transporters than the single treatment. To confirm these results, we measured the effects of STAT3 and NOTCH1 siRNAs on the expression of ABC transporters in CD109-overexpressing A2780 cells. Transfection with STAT3 siRNA reduced the activation of NOTCH1 and the expression level of ABC transporters, consistent with the results using CPT. In addition, siRNA-mediated NOTCH1 knockdown slightly inhibited the expression of ABCB1, in contrast to the drastic inhibition of ABCG2 expression (Figure S4). Moreover, the triple combinatorial treatment of CPT, DAPT, and PTX inhibited cell viability more significantly than single or dual-combinatorial treatment (CPT + DAPT, PTX + CPT, and PTX + DAPT) of each drug (Figure 6C). These results suggest that CD109-induced STAT3 phosphorylation leads to NOTCH1 activation, which leads to the reciprocal inhibition of STAT3, and that CD109-induced drug resistance in ovarian cancer cells is regulated via the STAT3-NOTCH1 signaling axis (Figure 6D).

## 3. Discussion

CD109, which is expressed on the cell surface of various malignant tumors, is involved in promoting tumor initiation, growth, and metastasis and is related to poor prognosis [9,24,25,26]. This study aimed to determine whether CD109 regulates drug resistance in EOC cells. Although the loss of CD109 in pancreatic ductal adenocarcinoma and cervical squamous cell carcinoma suppressed in vivo tumor growth [27,28], knockdown in A2780-R cells had no significant effect on transplanted tumor growth through cell proliferation (Figure 4 and Figure S5). Moreover, it has been reported that CD109 expression was positively correlated with chemotherapeutic resistance. CD109-positive cells sorted from glioma or triple-negative breast cancer show resistance to chemotherapy compared with CD109-negative cells [29,30]. Furthermore, CD109 knockdown in lung adenocarcinoma reduced chemotherapy resistance and inhibited tumor metastasis in mice [14]. We have previously reported that CD109 expression levels in tumors of EOC patients who relapsed after chemotherapy were 2.89 times higher than those in patients who did not relapse [16]. These results are consistent with our observation that CD109 expression is increased in A2780-R cells that have acquired drug resistance and that the CD109 expression level and drug resistance are positively correlated in vitro and in vivo. Therefore, targeted therapy with CD109 will be useful for the treatment of chemotherapy-resistant ovarian cancer. 

CD109 has been reported to promote tumor malignancy by regulating various signaling pathways. CD109 is known to induce tumor malignancy and poor prognosis through transforming growth factor beta (TGF-β) signal activation [25,31]. Furthermore, CD109 maintains cancer stem-like properties or promotes tumorigenicity and metastasis by interacting with EGFR in lung adenocarcinoma, cervical squamous cell carcinoma, and HNSCC [14,28,32]. In addition, Yes-associated protein (YAP) and transcriptional coactivator with PDZ-binding motif (TAZ) signaling regulated by CD109 upregulate tumor initiation and the radioresistance of glioma stem-like cells and promote epithelial–mesenchymal transition and stemness properties of lung adenocarcinomas, leading to metastasis [33,34]. The interaction between CD109 and GP130 is known to increase STAT3 phosphorylation, thereby promoting stemness properties, tumorigenicity, and chemoresistance of glioblastoma stem cells and metastasis of lung adenocarcinomas [15,26]. In the present study, we demonstrated, for the first time, that CD109 is involved in the activation of NOTCH1 signaling and STAT3 phosphorylation in A2780 cells. NOTCH1 signaling has been found to regulate chemoresistance in various cancers [35]. Qian et al. reported that NOTCH1 was increased in SKOV-3 EOC cells and that NOTCH1 mediates the increase in drug resistance [36]. *HES1*, the target gene of NOTCH1, showed a positive correlation with CD109. HES1 is associated with multidrug resistance by inducing the expression of ABC transporters [37,38]. Taken together, these results suggest that the CD109-induced activation of NOTCH1 plays a key role in the acquisition of drug resistance in EOCs.

In the present study, we demonstrated that the STAT3-NOTCH1 signaling axis activated by CD109 can modulate the drug resistance of A2780 cells. Moreover, the combined inhibition of the STAT3 and NOTCH1 signaling pathways significantly reduced the expression of ABC transporters and drug resistance. The coactivation of STAT3 and NOTCH1 is associated with cisplatin resistance in HNSCC, and the simultaneous downregulation of STAT3 and NOTCH1 using siRNA enhances chemosensitivity in breast cancer [21,22]. Koerdel et al. revealed that phospho-STAT3 stimulates the expression of the recombination signal binding protein for immunoglobulin kappa J (RBPJ), a major transcriptional effector of the NOTCH1 pathway, thereby promoting NOTCH signaling and increasing chemoradiotherapy resistance in gastrointestinal cancer [23]. It has been reported that STAT3 activity is amplified by HES1 in mouse neuroepithelial cells and colon cancer cells [39,40]. However, the present study demonstrated that STAT3 phosphorylation significantly increased upon the inhibition of NOTCH1 signaling activated by CD109 in A2780 cells, suggesting a negative feedback regulation of STAT3 activation by NOTCH1. These results suggest that the combined inhibition of both STAT3 and NOTCH1 is needed for the treatment of drug resistance in EOCs.

In summary, the present study demonstrated that CD109 expression is increased in drug-resistant A2780 cells, and CD109 acts as a switch that can regulate drug resistance. CD109 regulates drug resistance by activating the STAT3-NOTCH1 signaling axis. These results suggest that the CD109-STAT3-NOTCH1 signaling axis is a promising therapeutic target for the treatment of drug-resistant ovarian cancer.

## 4. Materials and Methods

### 4.1. Materials

Roswell Park Memorial Institute (RPMI) 1640, Dulbecco’s modified Eagle medium (DMEM), and trypsin–ethylene diamine tetraacetic acid (EDTA) solution were purchased from Welgene (Gyeongsan, Gyeongsangbuk-do, Republic of Korea). Fetal bovine serum (FBS), Hank’s balanced salt solution (HBSS), penicillin–streptomycin, and cell culture plates for adherent cells were purchased from Thermo Fisher Scientific, Inc. (Waltham, MA, USA). Antibodies against glyceraldehyde-3-phosphate dehydrogenase (GAPDH; sc-47724) and CD109 (sc-271085) were purchased from Santa Cruz Biotechnology (Dallas, TX, USA). Antibodies against ABCG2 (ab3380) were purchased from Abcam (Cambridge, UK). Antibodies against ABCB1 (#12683S), PARP-1 (#9542S), cleaved-PARP-1 (Asp214, #9541S), caspase-3 (#14220S), cleaved caspase-3 (Asp175, #9661S), STAT3 (#9139), p-STAT3 (Tyr705, #9131), NOTCH1 (#4380S), and cleaved NOTCH1 (Val1744, #4147) were purchased from Cell Signaling Technology (Danvers, MA, USA). The phycoerythrin (PE)-labeled mouse antihuman CD109 antibody (#556040) was purchased from BD Biosciences (Franklin Lakes, NJ, USA). 

### 4.2. Cell Culture

The human EOC cell line A2780 was maintained in the culture medium (RPMI1640 supplemented with 10% FBS and 1% penicillin-streptomycin). The drug-resistant A2780 (A2780-R) cell line was generated from the A2780 cells, as previously described [17]. Briefly, drug resistance was increased by subculturing A2780 cells with progressively increasing amounts of doxorubicin in the culture medium. The A2780-R cell line was also cultured under the same conditions as used for culturing the A2780 cells. HEK293FT cells used to produce lentiviruses for CD109 overexpression and knockdown were cultured in DMEM supplemented with 10% FBS and 1% penicillin-streptomycin.

### 4.3. Western Blot Analysis

Cells or tumor tissues were lysed using lysis buffer (20 mM Tris-HCl, 1 mM EDTA, 1 mM ethylene glycol tetraacetic acid,10 mM NaCl, 1 mM Na_3_VO_4_, 0.1 mM phenylmethylsulfonyl fluoride, 25 mM β-glycerol phosphate, 30 mM sodium pyrophosphate, and 1% Triton X-100; pH 7.4). Lysates were separated using sodium dodecyl sulfate–polyacrylamide gel electrophoresis and transferred onto nitrocellulose or polyvinylidene difluoride membranes. After blocking with 5% nonfat milk, membranes were immunoblotted with various primary antibodies. The bound antibodies were visualized with horseradish peroxidase (HRP)-conjugated secondary antibodies using an Advansta Western Bright ECL HRP Substrate Kit (Advansta Inc., San Jose, CA, USA).

### 4.4. Flow Cytometry Analysis

Flow cytometry analysis was performed to compare the expression levels of CD109 on the surface of A2780 and A2780-R cells. After cell counting, 10^6^ cells were resuspended in 100 μL of HBSS containing antihuman CD109-PE. The cells were stained in the dark for 30 min on ice and then washed twice with 1 mL HBSS. Cells labeled with CD109-PE were analyzed using an Attune NxT Acoustic Focusing Cytometer (Thermo Fisher Scientific, Inc.).

### 4.5. Overexpression and Knockdown of CD109

The following lentiviral vectors were used for the overexpression or knockdown of CD109: human CD109 lentiviral vector (pLV-EGFP:T2A:Puro-EF1A>hCD109 (NM_133493.5), VectorBuilder, Chicago, IL, USA), and human shCD109 lentiviral vector (TRCN0000073652). The shRNA target sequence was 5′-GCAGCCCTAATGAATACAGAA-3′. The HEK293FT cells were cotransfected with CD109 or shCD109 lentiviral vector, VSV-G envelope plasmid, and delta 8.9 packaging plasmid using a jetPRIME transfection reagent (Polyplus-Transfection, Illkirch, France). Subsequently, 48 h after transfection, virus-containing supernatant was added to the target cell culture medium along with polybrene (10 μg/mL) and incubated for 48 h. To obtain stable cells, lentivirus-infected cells were selected using puromycin (4 μg/mL). Green fluorescent protein and pLKO.1 puromycin-empty vectors were used as negative controls for CD109 and shCD109 lentiviral vectors, respectively.

### 4.6. Cell Viability Assay

Cell viability was measured by quantifying the amount of ATP in the culture medium and viable cells. The viability of cells under different treatment conditions was determined using the CellTiter-Glo^®^ Luminescent Cell Viability Assay (Promega, Madison, WI, USA), according to the manufacturer’s instructions. The A2780 and A2780-R cells were seeded at 10^4^ cells/well in 96-well culture plates and incubated for 24 h. After 48 h of incubation under various treatment conditions, a CellTiter-Glo^®^ Reagent was added at the same amount as the culture medium of each well. After cell lysis using an orbital shaker for 2 min, the luminescence signal was stabilized at room temperature for 10 min. The luminescence signal was measured using a VICTOR 3™ multilabel plate reader (Perkin Elmer, Waltham, MA, USA).

### 4.7. Tumorigenesis Assay in Xenograft Tumor Model

All animal studies adhered to protocols approved by the Pusan National University Institutional Animal Care and Use Committee. Six-week-old male BALB/c-nude (nu/nu) mice were purchased from Orient Bio, Inc. (Seongnam, Gyeonggi-do, Republic of Korea). The A2780-R (sh-con and -CD109) cells (5 × 10^5^ cells) were resuspended in 100 µL of Matrigel solution (1:1 dilution with HBSS) and injected subcutaneously into the right flank of the mice. After a week, tumor-bearing mice were intraperitoneally injected with PBS or 2 mg/kg PTX every 3 days, and the tumor appearance was inspected based on visual observation and palpation. The length (mm), width (mm), and height (mm) of tumor masses were measured using electronic Vernier calipers, and tumor volumes (mm^3^) were calculated as length × width × height × 0.52. Two weeks after the start of treatment, the mice were euthanized with CO_2_ gas, and the tumors were collected. The expression levels of CD109 and ABC transporters in all tumors were confirmed using Western blot analysis.

### 4.8. Knockdown via Small Interfering RNA (siRNA) Transfection

To transfect siRNA, the cells were seeded to 60–70% confluent in six-well culture plates. The cells were transfected with AccuTarget Predesigned siRNA specific for human STAT3 (si-STAT3 #1, 6774-1 and si-STAT3 #2, 6774-2) and NOTCH1 (si-NOTCH1 #1, 4851-1 and si-NOTCH1 #2, 4851-2) (BIONEER, Daejeon, Republic of Korea), siRNA specific for human CD109 (si-CD109; 5′-GACACUUACUCUUCCAUCATT-3′ and 5′-UGAUGGAAGAGUAAGUGUCTT-3′) or negative control siRNA (GenePharma, Shanghai, China) using the jetPRIME transfection reagent, according to the manufacturer’s instructions.

### 4.9. Quantitative Real-Time Polymerase Chain Reaction (PCR)

All RNA samples were extracted from cells using a TRIsure reagent (Meridian Bioscience, Cincinnati, OH, USA). Complementary DNA (cDNA) was synthesized from the mRNA samples (2 µg) using a reverse transcription system. Quantitative real-time PCR was performed using the QuantStudio™ 3 Real-Time PCR System (Applied Biosystems, Waltham, MA, USA) by mixing cDNA samples, primers of target genes, and SYBR Green PCR Master Mix (Thermo Fisher Scientific Inc.). The primer sequences used in this study were as follows: *GAPDH*, 5′-GGAGCCAAAAGGGTCATCAT-3′ and 5′-GTGATGGCATGGACTGTGGT-3′; *CD109*, 5′-AGATGCAAACCTCACGAAGG-3′ and 5′-ATGTGGACTGCTACCCAAAG-3′; *HES1*, 5′-CAACACGACACCGGATAAAC-3′ and 5′-AATGCCGCGAGCTATCTTTC-3′; *HES5*, 5′-CATCAACAGCAGCATCGAGC-3′ and 5′-TGCTTCAGGTAGCTGACAGC-3′; *HEY1*, 5′-AAGTTGCGCGTTATCTGAGC-3′ and 5′-AGCGTAGTTGTTGAGATGCG-3′; *HEY2*, 5′-GCCATACAGATGCCGACAGA-3′ and 5′-CAGTTACCGAGCTGCCTTGA-3′; *CCND1*, 5′-GGCGGAGGAGAACAAACAGA-3′ and 5′-CTCCTCAGGTTCAGGCCTTG-3′.

### 4.10. Dual-Luciferase Reporter (DLR) Assay

The interaction between CD109 and NOTCH1 was evaluated using the DLR assay. The A2780 (vector and CD109) cells were seeded at 10^5^ cells/well in a 12-well culture plate and incubated for 24 h. The A2780 cells were cotransfected with the 4 × CSL (CBF/SuH/Lag-1)-luciferase (four-time repeating section of the *RBP-J* target sequence, CGTGGGAA, with the firefly luciferase gene) reporter plasmid along with the Renilla luciferase control reporter plasmid using the jetPRIME transfection reagent. After 24 h, the cells were treated with 25 μM DAPT and incubated for another 24 h. Luciferase activity was measured using a VICTOR 3™ multilabel plate reader with the Dual-Luciferase Reporter Assay System (Promega), according to the manufacturer’s instructions. Firefly luciferase activity was normalized to the Renilla luciferase activity. 

### 4.11. Cell Proliferation Assay

Cell proliferation was measured using an EZ-Cytox cell proliferation assay kit (DoGenBio, Seoul, Republic of Korea) based on the conversion of water-soluble tetrazolium salt to insoluble formazan through mitochondrial activity in live cells. The cells were seeded at a density of 1000 cells/well in a 96-well culture plate. An EZ-Cytox reagent was added to the culture medium every 24, 48, 72, and 96 h, followed by incubation for 2 h, and the absorbance was measured using SpectraMax M2 (Molecular Devices, San Jose, CA, USA) at 450 nm.

### 4.12. Statistical Analysis

All experiments were performed in triplicate, and all data are presented as mean ± standard deviation. Statistical significance was analyzed using a two-tailed unpaired Student’s *t*-test. Statistical significance was set at 0.05. All statistical analyses were performed using SigmaPlot 14.0 software (Systat Software, San Jose, CA, USA).

## Data Availability

The data underlying this article will be shared upon reasonable request by the corresponding author.

## Supplementary Materials

The supporting information can be downloaded at: https://www.mdpi.com/article/10.3390/ijms241210306/s1.
