# Supplementary material for: CD109 Promotes Drug Resistance in A2780 Ovarian Cancer Cells by Regulating the STAT3-NOTCH1 Signaling Axis
Source: Int J Mol Sci. 2023 Jun 18;24(12):10306. doi: 10.3390/ijms241210306 (PMC10299649; doi:10.3390/ijms241210306)
Supplement: Supplementary file 1 [file ijms-24-10306-s001.zip › ijms-2340543-supplementary.pdf]

International Journal of Molecular Sciences

MDPI

Supplementary figures

CD109 Promotes Drug Resistance in A2780 Ovarian Cancer Cells by Regulating the STAT3-NOTCH1 Signaling Axis

Jun Se Kim [1], Min Joo Shin [1], Seo Yul Lee [1], Dae Kyoung Kim [2], Kyung-Un Choi [3], Dong-Soo Suh [4], Dayea Kim [5] and Jae Ho Kim [1,*]

[1] Department of Physiology, School of Medicine, Pusan National University, Yangsan 50612, Republic of Korea
[2] Hicelltech Inc., Yangsan 50612, Republic of Korea
[3] Department of Pathology, School of Medicine, Pusan National University, Yangsan 50612, Republic of Korea
[4] Department of Obstetrics and Gynecology, School of Medicine, Pusan National University, Yangsan 50612, Republic of Korea
[5] New Drug Development Center, Daegu-Gyeongbuk Medical Innovation Foundation (K-MEDI hub), Daegu 41061, Republic of Korea
* Correspondence: jhkimst@pusan.ac.kr

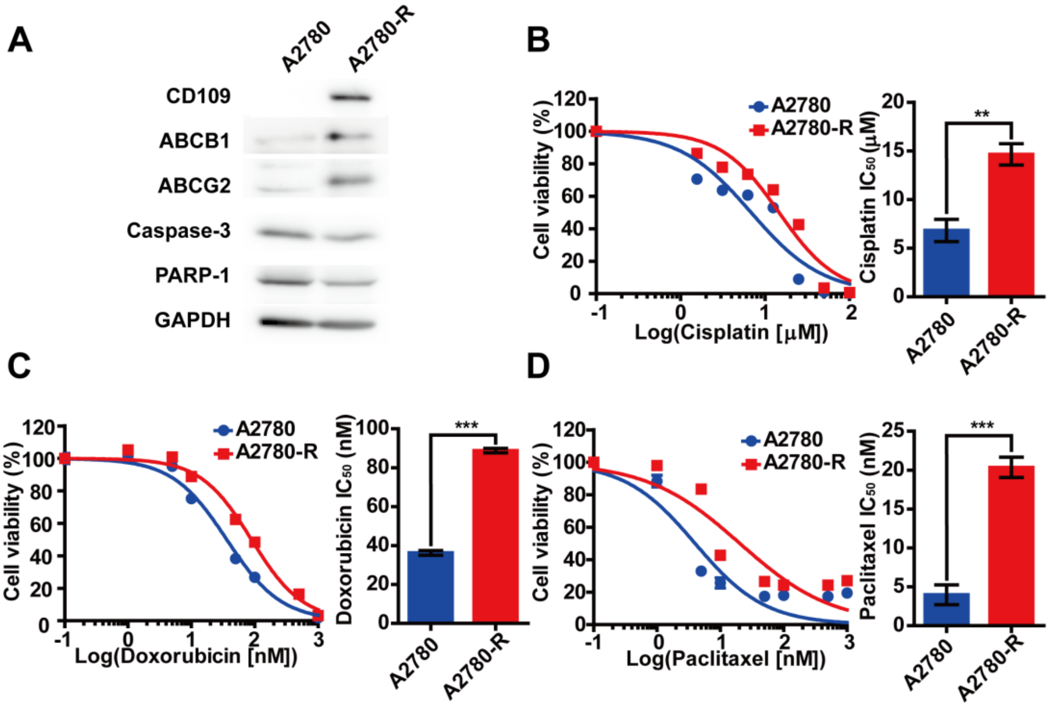

Figure S1. Multi-drug resistance of A2780-R cells. (**A**) Western blot analysis of CD109, ABC transporters (ABCB1 and ABCG2) and apoptosis markers (caspase-3 and PARP-1) in A2780 and A2780-R cells. parental A2780 and A2780-R cells were treated with the increasing doses of cisplatin (B), doxorubicin (C), and paclitaxel (D), followed by measurement of cell viability. Bar graphs show the IC_{50} values for cisplatin, doxorubicin, and paclitaxel in A2780 and A2780-R cells (**, P<0.01, ***, P< 0.001).

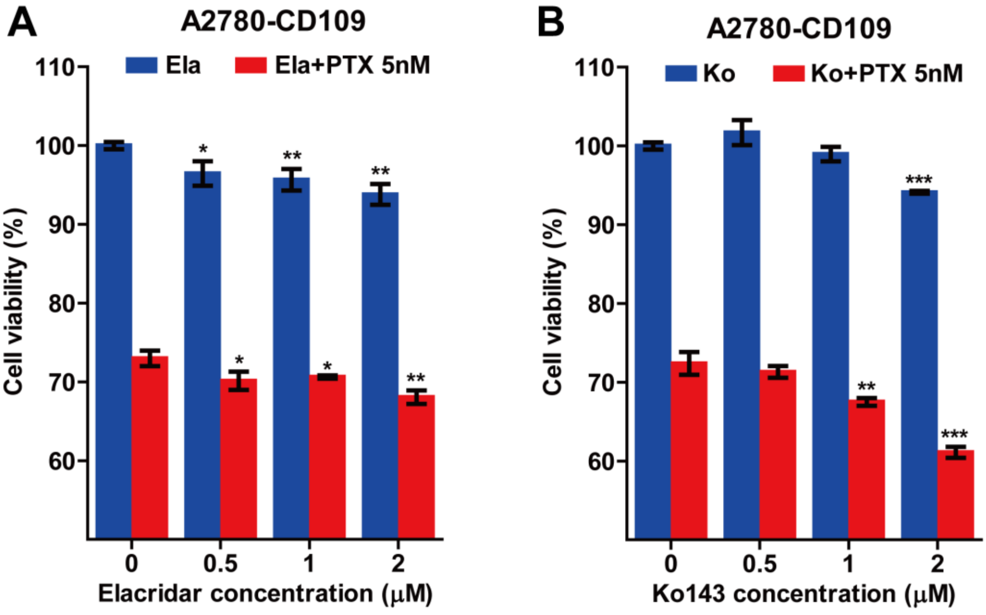

Figure S2. Effects of ABCB1 and ABCG2 inhibitors on resistance of CD109-overexpressed A2780 cells to paclitaxel. Dose-dependent effects of the ABCB1 inhibitor Elacridar **(A)** or the ABCG2 inhibitor Ko143 **(B)** on resistance of CD109-overexpressed A2780 cells to paclitaxel (PTX). The cells were treated with the increasing doses of Elacridar or Ko143 in the absence or the presence of 5 nM PTX, followed by measurement of cell viability. **, P<0.01, ***, P< 0.001 *vs* control.

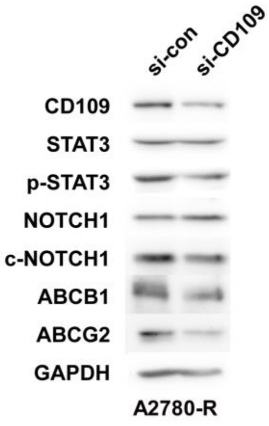

Figure S3. Effects of siRNA-mediated knockdown of CD109 on the STAT3-NOTCH1 signaling and the expression of ABC transporters. A2780-R cells were transfected with control siRNA (si-con) or CD109 siRNA (si-CD109), followed by western blot analysis of the STAT3-NOTCH1 signaling and the expression ABC transporters.

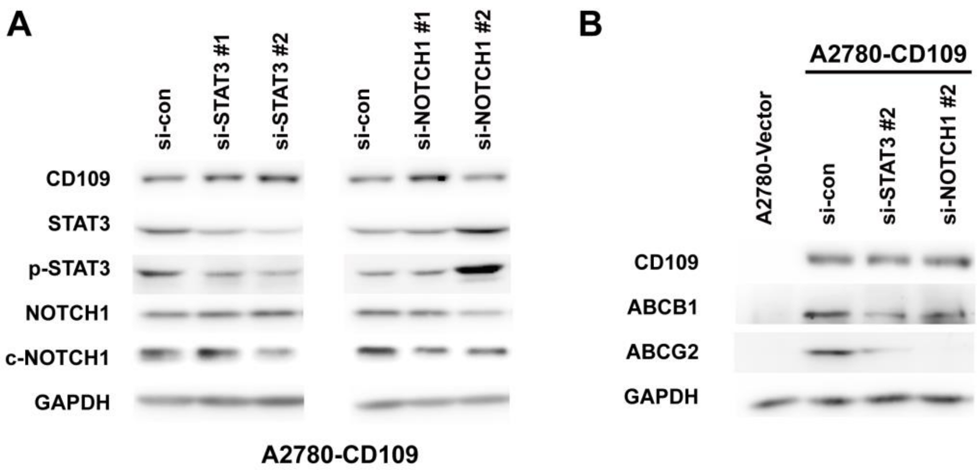

Figure S4. Effects of siRNA-mediated knockdown of STAT3 and NOTCH1 on the expression of ABC transporters in CD109-overexpressed A2780 cells. **(A)** Identification of siRNA clones specific to STAT3 or NOTCH1. CD109-overexpressed A2780 cells were transfected with two separate siRNA clones against STAT3 or NOTCH1, followed by Western blot analysis of STAT3 and NOTCH1 signaling. **(B)** The CD109-overexpressed A2780 cells were transfected with control siRNA, STAT3 siRNA #2 or NOTCH siRNA #2, followed by measurement of the expression levels of CD109, ABCB1, ABCG2, and GAPTH.

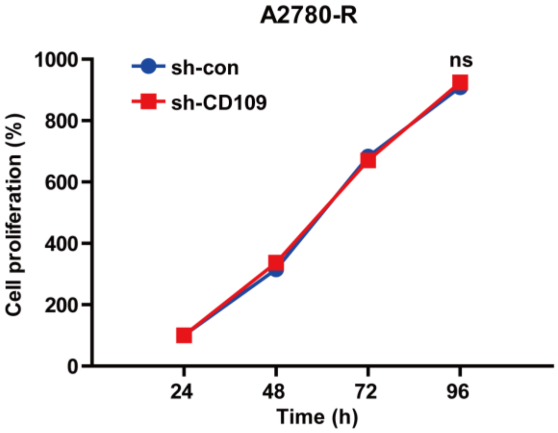

Figure S5. Identification of cell proliferation of A2780-R sh-con and sh-CD109 cells. CD109 knockdown showed no significance (ns) in the proliferation of A2780-R cells.
